# Mitochondrial genomes of stick insects (Phasmatodea) and phylogenetic considerations

**DOI:** 10.1371/journal.pone.0240186

**Published:** 2020-10-06

**Authors:** Nan Song, Xinghao Li, Risong Na

**Affiliations:** College of Plant Protection, Henan Agricultural University, Zhengzhou, China; Universite de Lausanne Faculte de biologie et medecine, SWITZERLAND

## Abstract

Phasmatodea represents an order of hemimetabolous insects. This group includes species with extreme forms of masquerade crypsis, whereby they imitate twigs, bark, lichen, moss, and leaves. In this study, we sequenced and annotated three mitochondrial genomes (mitogenomes) from Phasmatodea. The lengths of the novel mitogenomes range from 14,162 bp to 15,879 bp. The gene content and organization correspond to those inferred for the ancestral insect. We conducted phylogenetic analyses together with the existing mitogenomes of polyneopterans and mayflies. In most cases, the Phasmatodea was non-monophyletic, with Embioptera and Zoraptera nested inside. The mitogenome sequences from Embioptera and Zoraptera suffered from high substitution rates and displayed very long branches in phylogenetic trees. The monophyletic Phasmatodea was recovered only when the analysis employed the site-heterogeneous CAT-GTR model in PhyloBayes and used the nucleotide dataset PCG_nt. The Euphasmatodea was well established by various data types and inference methods. In addition, the clade Heteropterygidae and the subfamilies Lonchodinae and Necrosciinae were strongly supported. The Australasian clade Lanceocercata was recovered across analyses. However, the Clitumninae was non-monophyletic.

## Introduction

Phasmatodea represents an order of hemimetabolous insects, which are well-known as stick and leaf insects. They mimic sticks and leaves remarkably. Some phasmid species are the heaviest and largest extant insects [1]. For example, *Phobaeticus chani* is currently considered to be the longest extant insect, with a body length up to 570 mm [2]. Concerning insect biodiversity, the Phasmatodea is a comparatively small insect order including approximately 3,000 extant species classified in more than 480 genera [3, 4]. The phylogenetic relationships of this group remain contentious.

The monophyly of Phasmatodea is well supported by morphological traits [5, 6] and molecular evidence [7–9]. Nevertheless, the assumption of monophyletic Phasmatodea has also been challenged by some authors [10, 11]. Traditionally, Phasmatodea were divided into two groups: “Areolatae” (Pseudophasmatinae) and “Anareolatae” (Diapheromerinae), on the basis of the presence or absence of a triangular field at the apex of the tibiae [12–16]. However, this arrangement has never been supported by the phylogenetic analyses [3]. Tilgner (2002) [17] recognized both Areolatae and Anareolatae as being non-monophyletic based on morphological characters. Mitochondrial phylogenomic analyses have yet not corroborated the monophyly of both groups [18–20].

The wingless Nearctic walking-stick genus *Timema* is often recovered as the sister group to the remaining phasmids (the Euphasmatodea) [3, 5, 8, 9, 17–19, 21–28]. In contrast, some authors considered the *Timema* as a separate lineage [6, 18, 20]. Zompro (2004) [6] classified Phasmatodea into two suborders, namely Agathemerodea and Verophasmatodea. The Agathemerodea contains the sole family Agathemeridae, while the Verophasmatodea includes all other recent phasmids and the extinct Archipseudophasmatidae [29]. A more recent molecular analysis of transcriptome data retrieved three major clades of extant Phasmatodea, namely Timematodea (*Timema*), Aschiphasmatodea (Aschiphasmatinae), and Neophasmatodea (all remaining Phasmatodea) [9].

The phylogenetic placement of Phasmatodea in Polyneoptera is another highly controversial issue. Polyneoptera is an assemblage including 10 insect orders, namely, the Dermaptera, Embioptera, Grylloblattodea, Mantodea, Blattodea (including termites), Orthoptera, Plecoptera, Zoraptera, Mantophasmatodea and Phasmatodea [30]. Each given order within Polyneoptera was once presumed to be the sister group of Phasmatodea. In recent years, some molecular studies identified Embioptera as the closest relative of Phasmatodea [8, 30–34]. The sister-group relationship between Phasmatodea and Embioptera was supported by some morphological studies [35, 36]. Phasmatodea and Embioptera constituted a monophyletic clade named Eukinolabia [7]. However, other morphological data supported Phasmatodea as the sister group of Orthoptera [37, 38]. In addition, some authors suggested a close relationship between Mantophasmatodea and Phasmatodea [39].

Currently, only 20 mitogenomes from Phasmatodea have been sequenced (GenBank Apr. 30, 2020). Further mitogenomic data and broader taxon sampling are needed to elucidate the phylogeny of Phasmatodea. In the present study, we sequenced and annotated three mitogenomes from Necrosciinae (*Micadina brachptera*), Lonchodinae (*Phraortes* sp.) and Phasmatinae (Pharnaciini spec. indet.). Combined with the existing mitogenome sequences of 66 polyneopterans and two mayflies, we reconstructed the phylogenetic relationships in Polyneoptera, with emphasis on Phasmatodea.

## Materials and methods

### Sampling

Three stick insects, *M*. *brachptera*, *Phraortes* sp. and Pharnaciini spec. indet., were collected in July 2016, from Guangshui (31.86°N, 113.94°E), Hubei province, China. The adult samples were directly killed and fixed in 95% ethanol. The collected specimens were deposited at -20°C until DNA extraction. The taxonomy of the sequenced species is based on morphological characters, and on blasting matches to mitochondrial *cox1* records from the BOLD database (http://www.boldsystems.org/) and NCBI GenBank (http://www.ncbi.nlm.nih.gov/genbank/).

### Genome data generation and assembly

DNA extraction, Illumina sequencing, and reads filtering were conducted as previously described in [40]. *De novo* assembly for high-quality clean reads was performed using IDBA-TRAN [41]. The assemblies were constructed with the following parameter settings: 200 for the minimum size of contig, and 41 for an initial k-mer size, 10 for an iteration size, and 91 for a maximum k-mer size.

### Mitogenome reconstruction and annotation

Mitogenome reconstructions mostly followed a bioinformatics pipeline in [42]. We firstly created the BLAST databases with the assemblies from IDBA-TRAN [41]. The mitochondrial contigs corresponding to the stick insects were identified by BLAST search against the prior-sequenced bait gene fragments (*cox1*, *cob* and *rrnS*). The sequences of the oligonucleotide primers used for the determination of bait genes are listed in S1 Table.

The preliminary mitogenome annotations were conducted in the MITOS [43] webserver, under the reference of “RefSeq 63 Metazoa” and the “Invertebrate genetic code”. The gene boundaries were further checked and refined by alignment with homologous sequences of published phasmid species (see details in S2 Table) in MEGA 7 [44]. Mappings of the mitochondrial reads were proceeded with BWA v. 0.7.5 [45]. The reads were considered individually and not as pairs [46]. The SAM output was converted to a sorted BAM file by the program SAMtools v. 0.1.19 [47]. Statistics for nucleotide coverage were generated with Qualimap v.2.2.1 [48]. The classification information and accession numbers of the new mitogenome sequences are shown in S2 Table, and the sequence files under GenBank format are available in S1 File.

### Multiple sequence alignments

Protein-coding genes were translated into amino acid sequences based on the invertebrate mitochondrial genetic code, and aligned separately by using the MUSCLE [49] algorithm as implemented in MEGA 7 [44]. The alignment was back-translated into the corresponding nucleotide sequences. Each alignment was visually inspected, including manual removal of stop codons. Moreover, ambiguously aligned sites were removed through Gblocks 0.91b [50], with options for a “less stringent selection”. Finally, alignments were concatenated by using FASconCAT_v1.0 [51] to construct the amino acid dataset PCG_aa and the nucleotide dataset PCG_nt, respectively. Ribosomal and transfer RNA genes were aligned individually by using the program MAFFT under the iterative refinement method of “E-INS-i” [52]. The alignments were checked in MEGA 7 [44], and ambiguously aligned positions were excluded with Gblocks 0.91b [50] under the less stringent selections. Finally, all nucleotide alignments were concatenated together to compile the dataset PCGRNA (including 13 protein-coding genes, two rRNA genes and 22 tRNA genes), with FASconCAT_v1.0 [51]. All alignments used for the phylogenetic analyses are available in S2 File.

We used the yn00 program in the PAML package [53] to calculate the nonsynonymous (*dN*) and synonymous (*dS*) substitution rates of the concatenated protein-coding genes, with the method of [54]. DAMBE 7 [55] was used to conduct tests for substitution saturation of each data partition. Multiple sequence alignments were statistically scored by using AliStat [33]. Nucleotide compositions of the mitogenome sequences were estimated with MEGA 7 [44].

### Phylogenetic reconstructions

A total of 69 species representative of ten orders in Polyneoptera were included to constitute the ingroup taxa. Of which, 22 species represent nine subfamilies of Euphasmatodea and one represents the *Timema* (Timematidae). In addition, two species of Ephemeroptera were chosen as outgroups to root the polyneopteran tree.

Phylogenetic reconstructions were performed based on the datasets PCG_aa, PCG_nt and PCGRNA, with both maximum likelihood (ML) and Bayesian inferences (BI). Partitioned ML analyses were carried out with IQ-TREE v.1.6.10 [56]. We partitioned the matrices by genes, and used the partition schemes and the corresponding best-fit models (S3 Table) as designated by PartitionFinder 2 [57]. Branch support was evaluated with 30,000 ultrafast bootstrap replicates. BI analyses were performed on the same datasets with MrBayes v3.2.6 [58]. We used the MrBayes blocks generated by PartitionFinder 2 [57] for each of MrBayes analyses. We ran MrBayes by using four runs of Markov chain Monte Carlo (MCMC) chains for 10 million generations. We checked the convergence of the runs by the program Tracer 1.7 [59]. Trees were sampled every 1000, and the first 25% were discarded as burn-in.

In order to reduce the impact of long-branch attraction, we also used PhyloBayes-MPI [60] to conduct the BI analyses. The CAT-GTR model was applied to the analyses on the nucleotide datasets PCG_nt and PCGRNA, and the CAT-MTZOA model to the analysis on the protein dataset PCG_aa. For each PhyloBayes analysis, two runs with two chains each were run for 20,000 generations, and started from a random topology, respectively. The programs of *bpcomp* and *tracecomp* implemented in PhyloBayes package were used to check convergence of the chains. When the bipartition (maxdiff) values are less than 0.1 and all effective sizes are larger than 100, good runs are considered to be attained. Trees sampled after the burn-in from the two runs were combined and used to build the majority rule consensus tree.

### Hypothesis testing

In order to assess potential information contained in the dataset and to test for the alternative hypotheses of phylogenetic relationships among the polyneopteran orders, we carried out four-cluster likelihood mapping (FcLM) analyses [61] based on the datasets PCG_aa, PCG_nt and PCGRNA in IQ-TREE v.1.6.10 [56]. The partition schemes and the corresponding best-fit models were the same as those in the ML phylogenetic reconstruction. We binned species into four clusters: (1) Plecoptera (5 species), (2) Orthoptera (14 species), (3) Phasmatodea (23 species), and (4) Dictyoptera (22 species). The remaining species were included in the IGNORED cluster.

## Results

### Assembling mitogenomes

The Illumina sequencing yielded 81,772,168 paired-end 150 bp reads for the library containing Pharnaciini spec. indet., 64,299,118 for *Phraortes* sp., and 63,221,855 for *M*. *brachptera*, respectively. After filtering, 81,189,879 clean reads were obtained for the library containing Pharnaciini spec. indet., 58,984,030 for *Phraortes* sp., and 57,181,127 for *M*. *brachptera*. The mtDNA sequence of *M*. *brachptera* was assembled into a single scaffold with a length of 15,879 bp. A gap was detected in the control region. In addition, there were 28 bp and 26 bp missing nucleotides in the *cox1* and *cox2* genes, respectively. Each of Pharnaciini spec. indet. and *Phraortes* sp. was identified in two separate contigs. The mitogenome of Pharnaciini spec. indet. had a length of 15,192 bp, with two gap regions identified. One gap occurred in the control region, another was present in the *nad2* gene. Alignment with other phasmid species showed a 12-nucleotides missing sequence in *nad2*. The mitogenome of *Phraortes* sp. was 14,162 bp length, which contained three gap regions. The largest gap was located between *rrnS* and *nad2*. The second gap occurred in the *atp6* gene, which contained a 147-bp missing sequence. The third gap was present in the *nad1* gene, where a 44-bp missing sequence was identified as compared with other phasmid insects.

The coverage analysis of the mitogenome sequences demonstrated that the distribution of reads was not uniform across the mitogenome. There were sharp declines in the *nad2*, *nad4l*, *nad6* genes and the gap sequence of the control region. The mean coverage values were 70-fold for the mitogenome of *M*. *brachptera*, 53-fold for that of Pharnaciini spec. indet., and 60-fold for that of *Phraortes* sp.. The statistics for the Illumina sequencing of protein-coding genes and rRNA genes are shown in Table 1. For the same gene, different species had the similar coverage values. The *cox1*-*3* and *cob* genes had a greater coverage than other genes.

**Table 1:** Statistics for the assembling of protein-coding genes and rRNA genes of three new mitogenomes.

Genes	*Micadina brachptera*		Pharnaciini spec. indet.		*Phraortes* sp.	
	Mapped bases	Mean coverage	Mapped bases	Mean coverage	Mapped bases	Mean coverage
*nad2*	28050	28	4500	5	600	2
*cox1*	241952	185	239400	156	248105	161
*cox2*	47543	69	49650	72	49501	72
*atp6*	49651	73	11250	17	33451	54
*cox3*	90302	114	64651	82	26249	35
*nad3*	12450	35	13200	38	13201	38
*nad5*	138012	81	78150	45	80101	47
*nad4*	74707	56	50700	38	61050	46
*nad4l*	900	3	3450	13	750	3
*nad6*	2850	6	1200	3	2401	5
*cob*	165450	146	103650	91	63751	56
*nad1*	25652	27	20700	22	41997	45
*rrnL*	24752	20	26772	22	54602	46
*rrnS*	21751	28	15000	20	30452	41

Note: The analyses from Qualimap did not yield the statistics on the *atp8* gene and each of *tRNA* genes, due to their short sequence lengths (< 150 bp).

**Fig 1:**
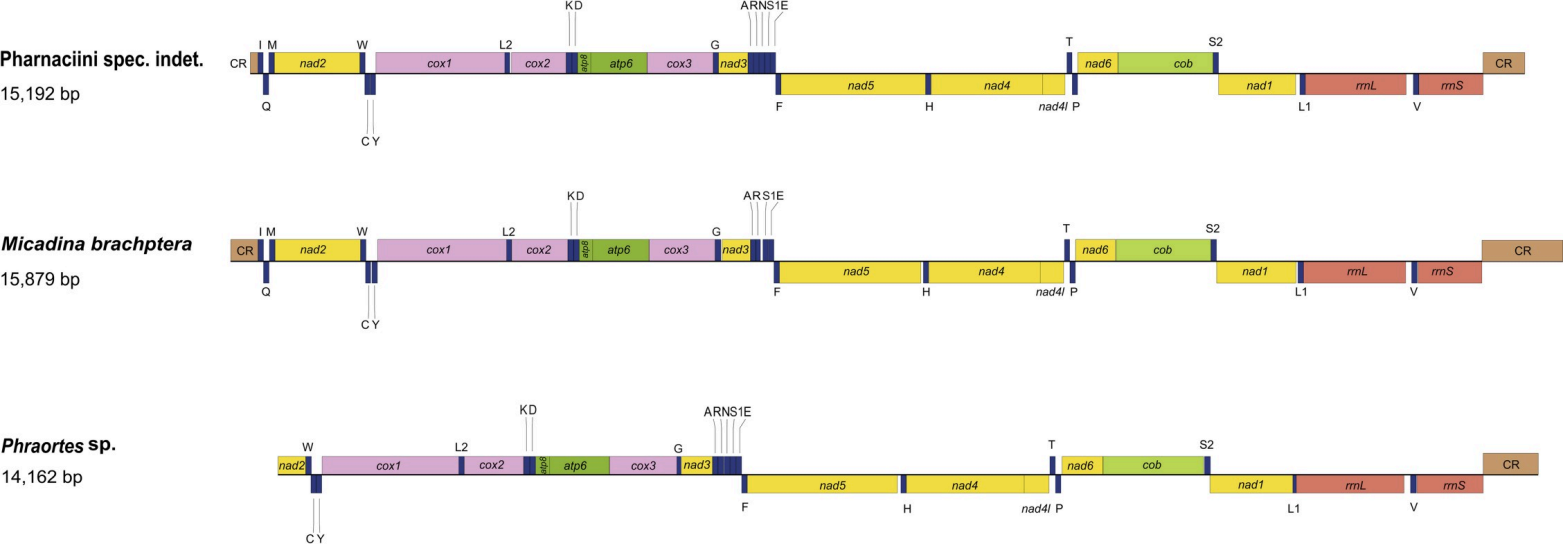
The structures of the mitochondrial genomes of Pharnaciini spec. indet., *Micadina brachptera* and *Phraortes* sp. The abbreviations of mitochondrial gene names are following those in MITOS webserver. The detailed annotations of the mitochondrial genomes are shown in S4 Table.

In the mitogenome of Pharnaciini spec. indet., we identified the typical 37 mitochondrial genes: 13 protein-coding genes, 22 tRNA genes and two rRNA genes (Fig 1 and S4 Table). The *trnN* was missing in *M*. *brachptera*, while the *trnI*, *trnM* and *trnQ* were missing in *Phraortes* sp.. In addition, the sequences were incomplete in the genes of *nad2* and *atp6* for *Phraortes* sp.. The gene organizations of the novel mitogenomes are consistent with the ancestral insect [62].

### Base composition and strand asymmetry

The A+T content of mitogenome was 78.0% for Pharnaciini spec. indet., 76.3% for *M*. *brachptera*, and 76.9% for *Phraortes* sp., respectively. These values were similar to those found in the published phasmatodean mitogenomes (mean A+T content of 76.4%). Distinct parts of the mitogenome displayed an A+T content that varied from 68.6% (*trnK*) to 92.4% (*trnE*) in Pharnaciini spec. indet., from 64.7% (*trnM*) to 92.3% (*trnE*) in *M*. *brachptera* and from 70.0% (*cox1*) to 89.2% (*trnE*) in *Phraortes* sp.. Protein-coding genes had an A+T content ranging from 74.7% (*M*. *brachptera*) to 79.0% (Pharnaciini spec. indet.), which was less than those in tRNA genes (*M*. *brachptera*: 77.8%, *Phraortes* sp.: 79.3%, and Pharnaciini spec. indet.: 80.0%). The A+T content of rRNA genes was 78.2% for *M*. *brachptera*, 76.7% for *Phraortes* sp., and 77.7% for Pharnaciini spec. indet..

The mean G+C content for the heavy (H) strand protein-coding genes ranged from 21.1% (Pharnaciini spec. indet.) to 24.9 (*M*. *brachptera*), while the mean G+C content for light (L) strand protein-coding genes was largely identical (21.1% for Pharnaciini spec. indet. and *Phraortes* sp. respectively, 21.5% for *M*. *brachptera*). We estimated GC-skew values [GC-skew = (G − C)/(G + C)] and obtained negative scores for all species’ protein-coding genes in the H-strand (-0.1392 for Pharnaciini spec. indet., -0.1448 for *M*. *brachptera*, and -0.1823 for *Phraortes* sp.). In contrast, all GC-skew values were positive for the L-strand protein-coding genes (0.0678 for Pharnaciini spec. indet., 0.2381 for *M*. *brachptera*, and 0.2622 for *Phraortes* sp.). The AT-skews [AT-skew = (A − T)/(A + T)] were contrary to the GC-skews in that H-strand had a positive AT-skew (0.0892 for Pharnaciini spec. indet., 0.1018 for *M*. *brachptera*, and 0.2520 for *Phraortes* sp.) and L-strand had a negative AT-skew (-0.3728 for Pharnaciini spec. indet., -0.3612 for *M*. *brachptera*, and -0.3703 for *Phraortes* sp.). The results indicated the asymmetric usage of four base pairs between the H- and L-strands, namely that G was preferentially located in the L-strand and A was richer in the H-strand.

### Codon usage

All 13 protein-coding genes used ATN (ATG, ATT or ATA) as an initiation codon. The most frequent termination codons used were TAA and TAG. The incomplete stop codon T was used in the genes *cox2* (Pharnaciini spec. indet., *M*. *brachptera* and *Phraortes* sp.), *nad3* (Pharnaciini spec. indet. and *M*. *brachptera*) and *nad5* (*M*. *brachptera*). In addition, the *nad5* gene in *Phraortes* sp. ended with the incomplete stop codon TA. The post-transcriptional polyadenylation is thought to create a complete TAA termination codon, as observed in other insects.

Codon usage analyses showed that ATA for methionine, ATT for isoleucine and TTA for leucine were the three most represented codons in the H-strand of the Pharnaciini spec. indet. mitogneome. TTT for phenylalanine, TTA for leucine and ATT for isoleucine were the three most represented codons in the L-strand. The mitogenomes of *Phraortes* sp. and *M*. *brachptera* had the same codon usage patterns as Pharnaciini spec. indet.. The codon usage patterns indicated that the mitogenomes were highly skewed towards codons with high A+T content.

### Transfer RNA and ribosomal RNA genes

All tRNA genes had the standard anticodons, and ranged in size from 62 bp to 70 bp. The inferred secondary structures for tRNA genes can be folded into canonical clover-leaf model, except for *trnS1* and *trnR*. With regard to the *trnS1* gene in *M*. *brachptera* and *Phraortes* sp., the dihydrouridine (DHU) arm formed a simple loop. In the *trnR* gene of *M*. *brachptera*, the TΨC arm was incomplete, with only a simple T loop structure inferred. All secondary structures for tRNA genes are presented in S1 Fig.

Two rRNA genes (*rrnL* and *rrnS*) were present in the novel mitogenomes, and these were located between *trnL1* and *trnV* and between *trnV* and the control region, respectively (Fig 1 and S4 Table). The lengths of *rrnL* genes ranged from 1,217 bp (*Phraortes* sp.) to 1,247 bp (*M*. *brachptera*), while *rrnS* ranged from 746 bp (*Phraortes* sp.) to 767 bp (Pharnaciini spec. indet.). The predicted rRNA secondary structures (S2 Fig) illustrated that the *rrnL* molecules contained five domains (labeled I, II, IV, V and VI; lacking domain III) and 44–45 helices, and the *rrnS* molecules were comprised of three domains (labeled I, II, III) and 27–29 helices.

### Substitution saturation and genetic divergence

The substitution saturation tests showed no significant level of saturation in the alignments of PCG_nt, trn and rrn (*Iss* < *Iss*.*cSym* and *Iss < Iss*.*cAsym*, S5 Table). There were substantial differences in *dN* values among polyneopteran groups (Table 2). The rate of sequence evolution of Embioptera was obviously higher than other lineages. In contrast, *dS* values were similar among groups. The ratio of nonsynonymous to synonymous substitutions (*dN*/*dS*) showed the same distribution pattern as *dN* values. Within Polyneoptera, the Embioptera, Zoraptera and Dermaptera had the relatively high *dN*/*dS* values.

**Table 2:** The non-synonymous (*dN*) substitutions and synonymous (*dS*) substitutions estimated by yn00 implemented in PAML.

Order	*dN*	*dS*	*dN/dS*
Blattodea	0.1815	4.1455	0.0438
Dermaptera	0.2676	5.3809	0.0497
Embioptera	0.3481	4.1151	0.0846
Ephemeroptera	0.1989	5.2814	0.0377
Grylloblattodea	0.1843	4.7984	0.0384
Mantodea	0.1963	5.1902	0.0378
Mantophasmatodea	0.2009	4.9451	0.0406
Orthoptera	0.1980	4.3637	0.0454
Phasmatodea	0.1965	4.5389	0.0433
Plecoptera	0.1889	4.9672	0.0380
Zoraptera	0.3521	4.1131	0.0856

### Phylogenetic analyses

**Fig 2:**
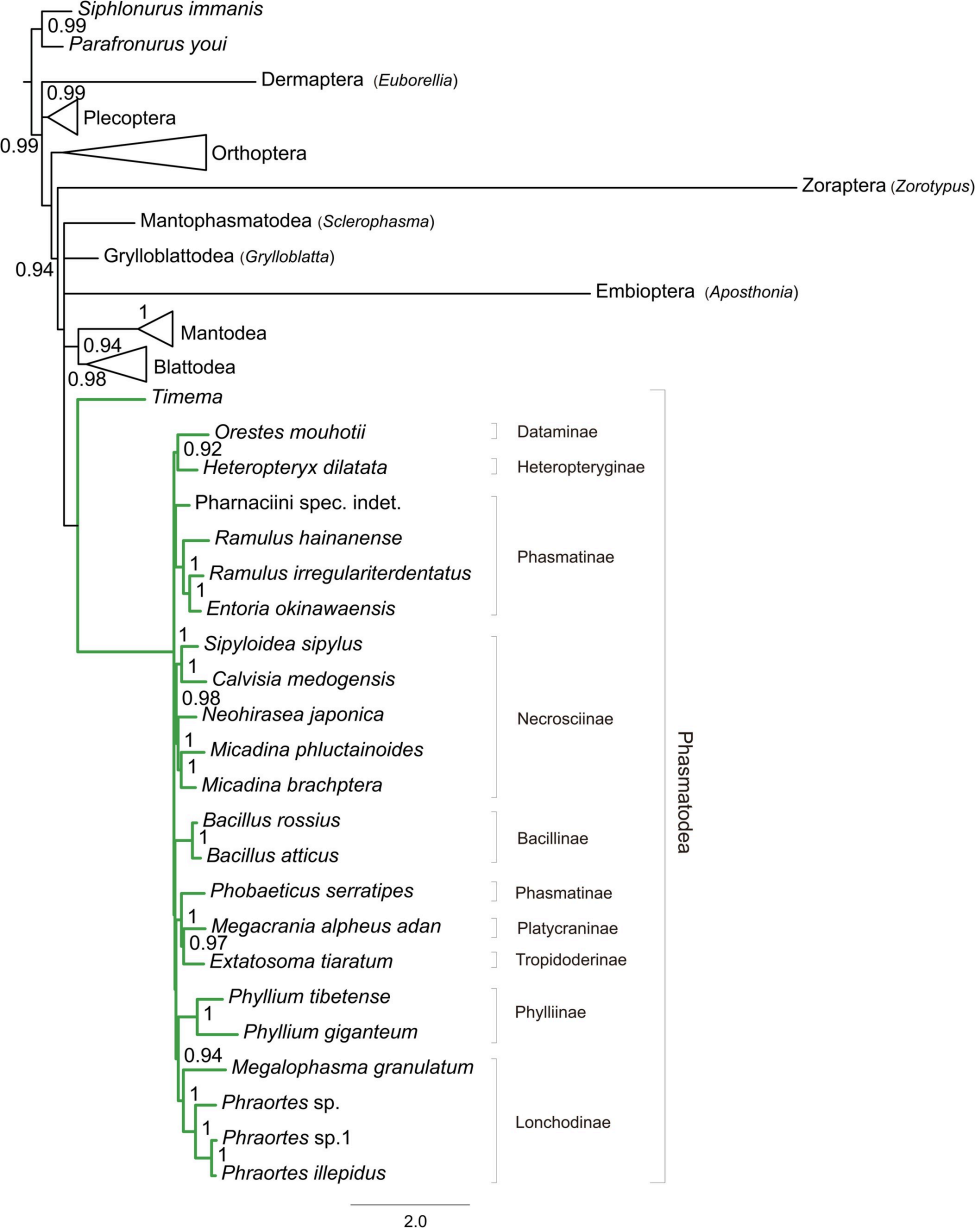
The simplified Bayesian tree inferred from the dataset PCG_nt by using PhyloBayes, under the site-heterogeneous CAT-GTR model. Node numbers show the poster probability values (≥ 0.9). Green lines indicate the Phasmatodea lineages. For the full tree, see S3C Fig.

In this study, a total of nine phylogenetic trees were reconstructed for the phylogeny of Polyneoptera (Fig 2 and S3–S5 Figs), and each resulted in somewhat different relationships. Current mitogenomic data supported the monophyly of Plecoptera, Orthoptera, Mantodea, and Blattodea. However, the Phasmatodea was not monophyletic in most cases, because the Embioptera + Zoraptera clade was the sister group to Euphasmatodea (S4 and S5 Figs). Within Polyneoptera, Mantodea and Blattodea formed the group Dictyoptera. Moreover, the sister-group relationship between Mantodea and Blattodea was strongly supported by all trees (BP = 100, PP > 0.94).

Tree topologies between analyses were different in three ways: (1) the relative branching order between the two early-diverging lineages of polyneopterans, namely Dermaptera and Plecoptera; (2) the placements of Orthoptera and Dictyoptera; (3) the monophyly of Phasmatodea.

Both Dermaptera and Plecoptera were supported as the earliest diverging lineages of Polyneoptera. But different data types resulted in the different branching sequence. The nucleotide datasets PCG_nt and PCGRNA placed Dermaptera as the most basal clade (Fig 2 and S3 and S4 Figs), whereas the amino acid dataset PCG_aa retrieved Plecoptera as the first splitting lineage (S5 Fig).

**Fig 3:**
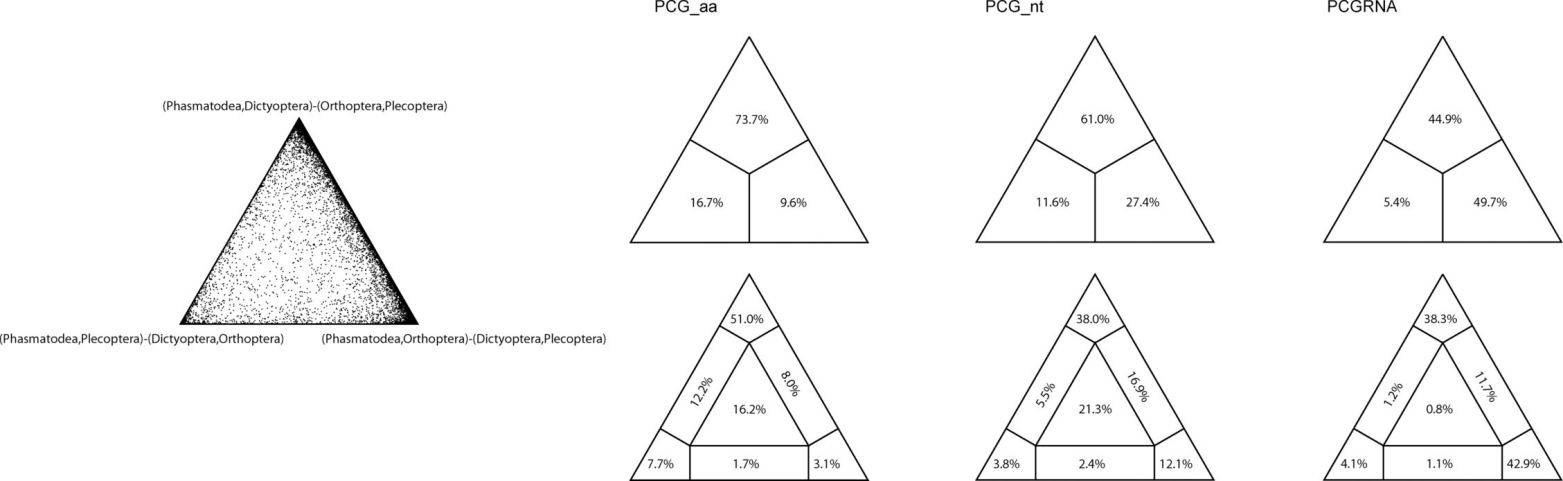
Results obtained from the four-cluster likelihood-mapping analysis showing support for the hypotheses of conflict nodes. The left triangle picture shows the possible relationships of four clusters as defined in the section of materials and methods. The above triangle pictures on the right are the three posterior probabilities for the three possible unrooted trees of four clusters from each dataset. The below triangle pictures on the right show the seven areas supporting different evolutionary information from each dataset.

In the ML analyses and the BI analyses under the homogeneous GTR model, data type influenced the placements of Orthoptera and Dictyoptera. The nucleotide datasets PCG_nt and PCGRNA more frequently recovered Orthoptera as the sister group of a clade including Phasmatodea, Embioptera and Zoraptera. The amino acid dataset PCG_aa presented a tree distinct from those on the nucleotide datasets, where the Orthoptera was placed in a more basal position, with Dictyoptera forming the sister group to a clade consisting of Phasmatodea. The FcLM analyses revealed a large amount of conflicting signals in the datasets. Only the amino-acid dataset PCG_aa showed weak support for the tree topology of (Phasmatodea + Dictyoptera) + (Orthoptera + Plecoptera) (Fig 3, 51.0% of quartets). The PhyloBayes analyses under the site-heterogeneous CAT-GTR or CAT-MTZOA models consistently recovered a close relationship of Dictyoptera to Phasmatodea, irrespective of the data type. The FcLM results when analyzing the nucleotide datasets showed some weaker signal for the branching pattern (Fig 3, 38.0% and 38.3% of quartets, PCG_nt and PCGRNA respectively).

The monophyly of Phasmatodea was only recovered by the dataset PCG_nt under the PhyloBayes inference using the site-heterogeneous CAT-GTR model (Fig 2 and S3C Fig). Although the PhyloBayes analyses on the datasets PCGRNA and PCG_aa (S4C and S5C Figs) did not support a monophyletic Phasmatodea, the single clade comprising the long-branched taxa (i.e., Zoraptera and Embioptera) has been distracted under the site-heterogeneous CAT-GTR or CAT-MTZOA model.

The monophyly of Euphasmatodea was well supported, although relationships among the constituent subfamilies varied across analyses. We found significant support for the monophyly of Lonchodinae and Necrosciinae. However, the Clitumninae was not monophyletic in all trees, with respect to the *Phobaeticus*. Three species, namely *Phobaeticus serratipes* (Phasmatinae), *Megacrania alpheus adan* (Platycraninae) and *Extatosoma tiaratum* (Tropidoderinae), were always clustered together in a single clade. Especially, the PhyloBayes trees resolved the branching order of (Phasmatinae + (Platycraninae + Tropidoderinae)). Several analyses placed *Orestes mouhotii* (Dataminae) as the closest sister group to *Heteropteryx dilatata* (Heteropteryginae), both of which formed the family Heteropterygidae. Four trees inferred from the datasets PCG_nt and PCG_aa under the homogeneous GTR model suggested that the Phylliinae was the most primitive subfamily in Euphasmatodea. In contrast, trees from the remaining analyses (PCGRNA-ML, PCGRNA-MrBayes, PCG_nt-PhyloBayes, PCGRNA-PhyloBayes and PCG_aa-PhyloBayes) recovered Phylliinae as a more derived clade. Most analyses showed that the Necrosciinae included the species situated at the tips of terminal branches.

In the PhyloBayes analyses, the newly determined species Pharnaciini spec. indet. (Phasmatinae) formed the sister group to the main clade of Clitumninae (Fig 2; S3C, S4C and S5C Figs). Other two species sequenced in this study can also be assigned to the group to related taxa unambiguously (i.e., *Phraortes* sp. in Lonchodinae, and *M*. *brachptera* in Necrosciinae). These results demonstrated that the present mitogenome data can be useful in resolving the lower level relationships of Phasmatodea.

## Discussion

The polyneopterans represent the relatively old groups of winged insects. They display complex features of lifestyles and external morphology across lineages. Although the phylogeny derived from the most recent analysis contains a range of nodes that are well supported (e.g., [30]), some relationships within Polyneoptera remain poorly resolved [63]. In this study, the Phasmatodea was in most cases retrieved to be a non-monophyletic assemblage, with respect to Embioptera and Zoraptera. In contrast, the analyses of transcriptomes [9, 30, 33] recovered a monophyletic Phasmatodea comprising *Timema* and Euphasmatodea. The non-monophyletic Phasmatodea returned by the present mitogenome data may be an artifact of long-branch attraction.

### The monophyly of Phasmatodea

Recent studies on the Phasmatodea phylogeny often supported the monophyly of this group [9, 19, 30]. But the studies of [18, 34] retrieved a non-monophyletic Phasmatodea based on the mitogenome sequence data. Most analyses in this study yielded a similar result as those in [18, 34]. Despite with this, some lines of evidence have shown that the clade Zoraptera + Embioptera clustering with Euphasmatodea is misplaced because of long-branch attraction. First, the evolutionary rates of different polyneopteran lineages were highly divergent, and it has been shown that the faster evolving Zoraptera and Embioptera tended to group together and form highly unstable long branches in phylogenetic trees. Second, the use of more realistic models of sequence evolution was also known to attenuate the impact of long-branch attraction, which led to a recovery of the monophyletic Phasmatodea (Fig 2 and S3C Fig) and the long-branch distraction (S4C and S5C Figs). The CAT-series models implemented in the program PhyloBayes were developed to account for the heterogeneous sequence evolution and reduce the negative effects of compositional and mutational bias [64–67]. Previous studies have demonstrated the power of these models in suppressing the long-branch attraction artefacts in the animal phylogeny [40, 65–69]. In the present study, the analyses also showed that the site-heterogeneous CAT-GTR and CAT-MTZOA models are significantly more robust against long-branch attraction, compared to the homogeneous GTR models. Therefore, the resultant trees from the PhyloBayes analyses under the site-heterogeneous CAT-GTR or CAT-MTZOA model more closely reflected the phylogenetic relationships of Phasmatodea and Polyneoptera.

### The phylogenetic relationships among stick and leaf insects

Within Euphasmatodea, different methods of analysis produced different branching patterns. The trees inferred for the Euphasmatodea phylogeny were characterized by a plethora of short internodes, which was consistent with the hypothesis of an early and rapid radiation of major phasmatodean lineages [3, 70].

The Phylliinae, or the true leaf insects, have an extreme form of morphological features, with a dorsoventrally flattened body and the broadly expanded legs, thus giving these insects a leaf-like appearance. Their morphological distinctiveness has led to suggestions that they might have an origin independent from the stick insects [1, 11, 71]. However, this view has recently been overturned by new morphological and molecular data [7–9, 22–24, 27, 34, 35, 72, 73]. More and more analyses have tended to place Phylliinae as a subordinate taxon within Euphasmatodea [9, 22, 23]. Fossil evidence also suggested that leaf insects descended from the stick insect-like ancestors [74]. However, the exact phylogenetic position of Phylliinae is still under debate [9, 18, 27, 73]. Different data and analysis yielded highly conflicting results [9, 72]. In this study, five out of nine analyses recovered Phylliinae as a more derived clade. This is consistent with the view that Phylliinae has a subordinate position within Euphasmatodea [9, 15].

Previous studies have indicated that the traditional anareolate subfamily ‘Phasmatinae’, comprising Clitumnini, Medaurini, Pharnaciini and Phasmatini, is a polyphyletic group [2, 23, 35]. Based on the morphological similarity, Hennemann and Conle (2008) [2] established the subfamily Clitumninae, comprising Clitumnini, Medaurini and Pharnaciini. The monophyly of Clitumninae was confirmed by the study of [9]. But many previous authors found Clitumninae to be polyphyletic [23, 24, 27, 73, 75, 76]. The present analyses consistently recovered a non-monophyletic Clitumninae. Because the *Phobaeticus* (Pharnaciini) was distantly related to the main clade of Clitumninae. Hennemann and Conle (2008) [2] suggested a close relationship between Lonchodinae and Clitumninae. But this hypothesis has not yet been corroborated by recent phylogenetic analyses [9, 27]. The current mitogenomic data offered no clear support for a stable placement of Clitumninae within Euphasmatodea.

The Australasian clade Lanceocercata is proposed by [77, 78], comprising genera from the subfamilies Tropidoderinae, Xeroderinae, “Pachymorphinae”, “Phasmatinae” and “Platycraninae”. In this study, two species representing Lanceocercata are included: *E*. *tiaratum* (Tropidoderinae) and *M*. *alpheus adan* (Platycraninae). Both species formed a monophyletic clade in most analyses.

## Conclusions

The three additional mitogenome sequences of stick insects presented in this study contribute to make sense of phasmatodean phylogeny. Our estimate of the Phasmatodea phylogeny largely supports the subfamilial classification previously proposed for this group. However, the non-monophyly of Phasmatodea reconstructed by most analyses conflicts with recent studies [30, 33]. The Zoraptera and Embioptera were the sister groups of Euphasmatodea, which rendered *Timema* as a separate lineage. This arrangement may be a consequence of long-branch attraction artifact, because the mitogenomes of Zoraptera and Embioptera have rapid rates of sequence evolution. Further refinement of gene sequences and analytical methods are needed to allow accurate estimation of phylogeny for Phasmatodea. Due to the data availability, the New World clade Occidophasmata and the subfamily Aschiphasmatinae are missing in this study. Future mitogenome studies should cover the species of both groups to comprehensively assess the phylogeny of the Euphasmatodea.

## Supporting information

S1 Fig The inferred tRNA secondary structures for the newly sequenced mitochondrial genomes. (A) Pharnaciini spec. indet., (B) *Micadina brachptera*, and (C) *Phraortes* sp.. Watson-Crick base pairs are indicated by lines, and wobble G-U base pairs are indicated by dots. The non-canonical base pairs are not marked.
(TIF)

S2 Fig The inferred rRNA secondary structures the newly sequenced mitochondrial genomes. (A-1) *rrnL* of Pharnaciini spec. indet., (A-2) *rrnS* of Pharnaciini spec. indet., (B-1) *rrnL* of *Micadina brachptera*, (B-2) *rrnS* of *Micadina brachptera*, (C-1) *rrnL* of *Phraortes* sp. and (C-2) *rrnS* of *Phraortes* sp.. Watson-Crick base pairs are indicated by lines, and wobble G-U base pairs are indicated by dots. The non-canonical base pairs are not marked. The numbers I, II, IV, V and VI represent the five domains in the *rrnL* gene. The numbers I–Ⅲ represent the three domains in the *rrnS* gene.
(TIF)

S3 Fig Phylogenetic trees from the dataset PCG_nt. (A) ML tree was reconstructed by IQ-TREE. The partition schemes and best-fitting models were selected by PartitionFinder. Node numbers show bootstrap support values. (B) Bayesian tree was reconstructed by MrBayes. The partition schemes and best-fitting models were selected by PartitionFinder. Node numbers show the poster probability values. (C) Bayesian tree was reconstructed by PhyloBayes. The CAT-GTR model were used in this analysis. Node numbers show the poster probability values.
(TIF)

S4 Fig Phylogenetic trees from the dataset PCGRNA. (A) ML tree was reconstructed by IQ-TREE. The partition schemes and best-fitting models were selected by PartitionFinder. Node numbers show bootstrap support values. (B) Bayesian tree was reconstructed by MrBayes. The partition schemes and best-fitting models were selected by PartitionFinder. Node numbers show the poster probability values. (C) Bayesian tree was reconstructed by PhyloBayes. The CAT-GTR model were used in this analysis. Node numbers show the poster probability values.
(TIF)

S5 Fig Phylogenetic trees from the dataset PCG_aa. (A) ML tree was reconstructed by IQ-TREE. The partition schemes and best-fitting models were selected by PartitionFinder. Node numbers show bootstrap support values. (B) Bayesian tree was reconstructed by MrBayes. The partition schemes and best-fitting models were selected by PartitionFinder. Node numbers show the poster probability values. (C) Bayesian tree was reconstructed by PhyloBayes. The CAT-MTZOA model were used in this analysis. Node numbers show the poster probability values.
(TIF)

S1 Table The primers used for amplifying and sequencing the bait genes. (XLSX)

S2 Table Taxa included in this study. (XLSX)

S3 Table A. The partitioning schemes and best-fitting modes selected by PartitionFinder for the dataset PCG_nt. B. The partitioning schemes and best-fitting modes selected by PartitionFinder for the dataset PCGRNA. C. The partitioning schemes and best-fitting modes selected by PartitionFinder for the dataset PCG_aa.
(XLSX)

S4 Table Organization of the newly determined stick insects’ mitogenomes. (XLSX)

S5 Table Substitution saturation tests conducted in DAMBE. (XLSX)

S1 File (ZIP)

S2 File (ZIP)
