# Supplementary figures and images for: Mitochondrial genomes of stick insects (Phasmatodea) and phylogenetic considerations
Source: PLoS One. 2020 Oct 6;15(10):e0240186. doi: 10.1371/journal.pone.0240186 (PMC7537864; doi:10.1371/journal.pone.0240186)

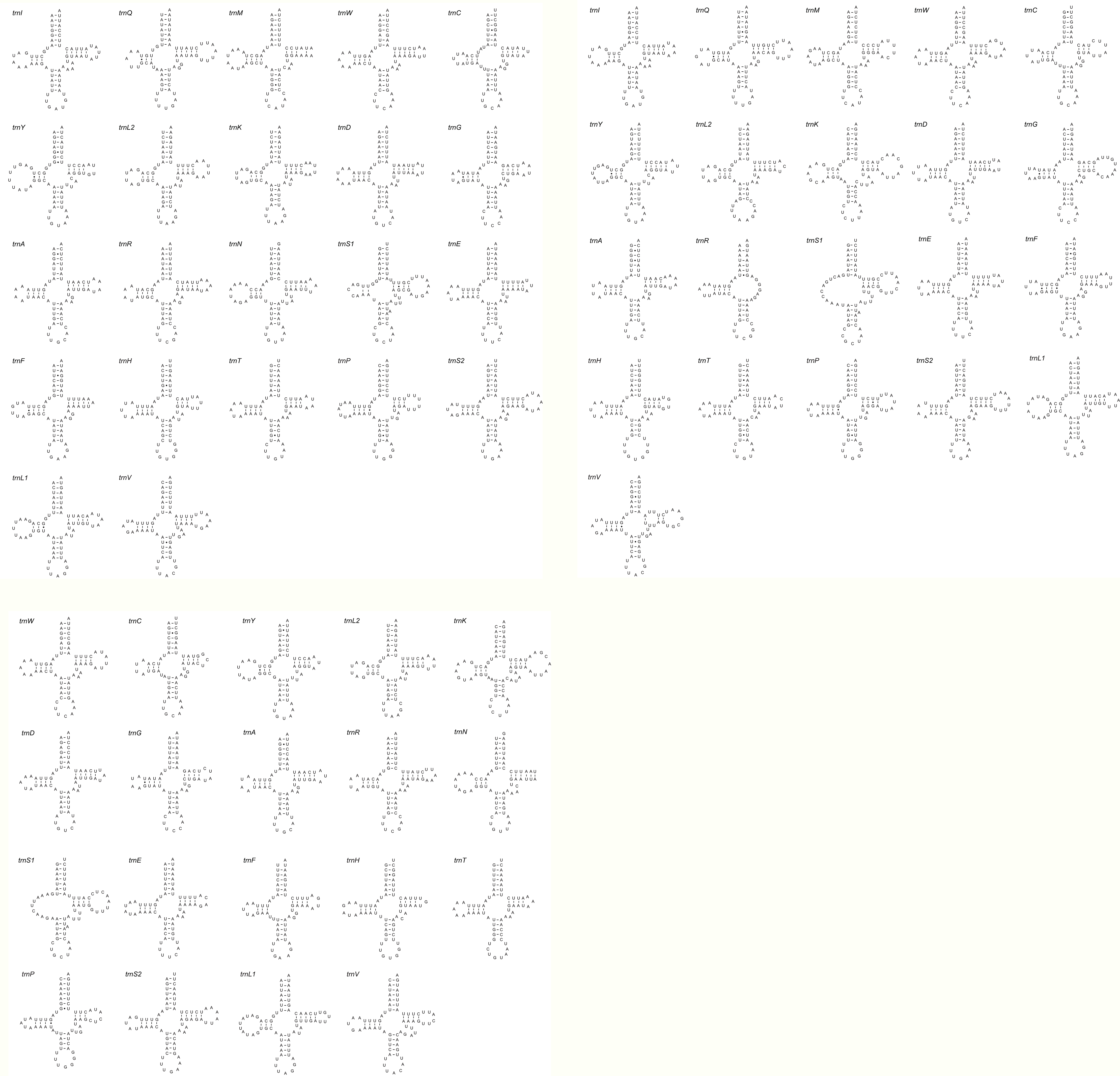

Supplement: S1 Fig — (A) Pharnaciini spec. indet., (B) Micadina brachptera, and (C) Phraortes sp.. Watson-Crick base pairs are indicated by lines, and wobble G-U base pairs are indicated by dots. The non-canonical base pairs are not marked. (TIF) [file pone.0240186.s001.tif]

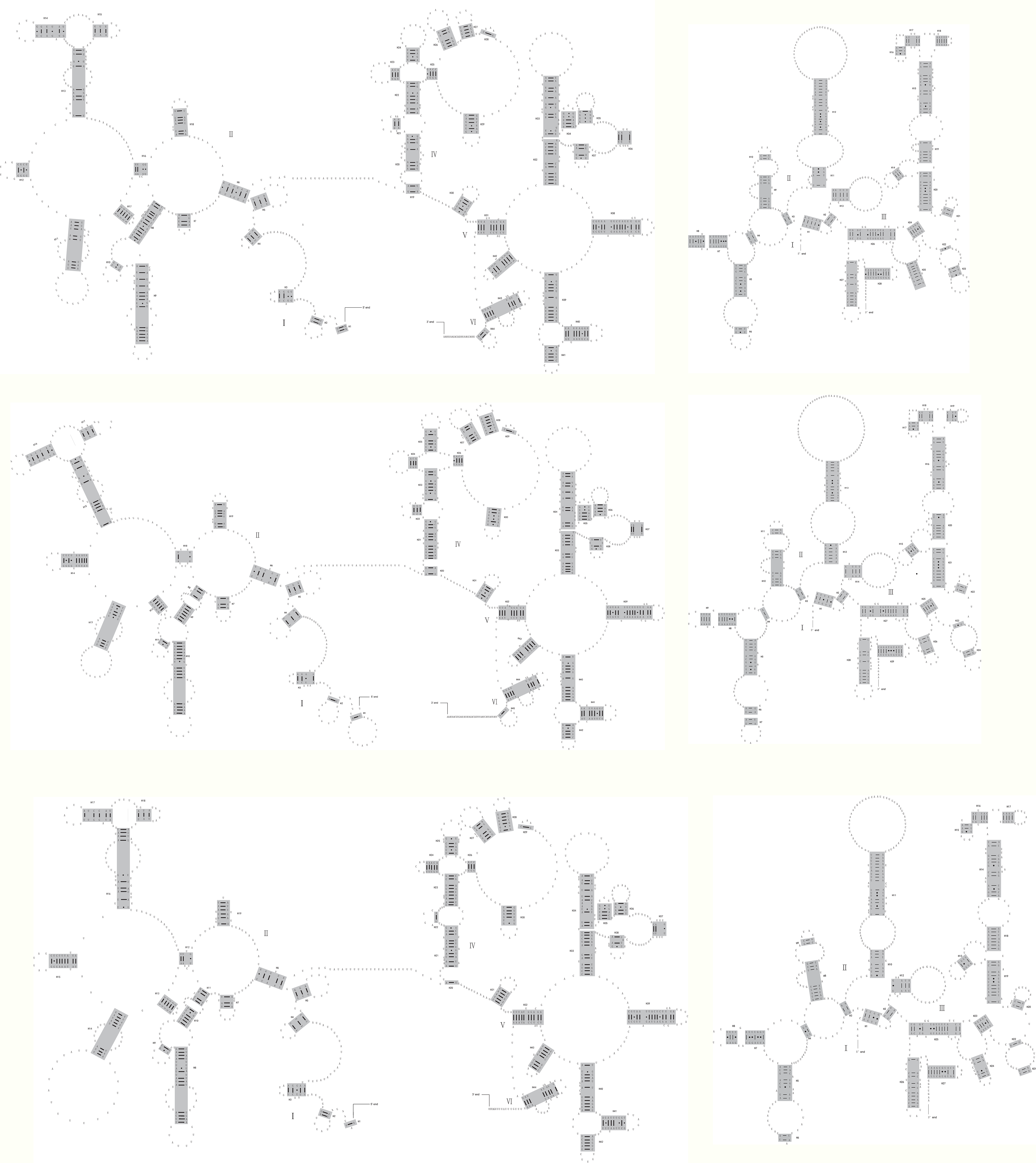

Supplement: S2 Fig — (A-1) rrnL of Pharnaciini spec. indet., (A-2) rrnS of Pharnaciini spec. indet., (B-1) rrnL of Micadina brachptera, (B-2) rrnS of Micadina brachptera, (C-1) rrnL of Phraortes sp. and (C-2) rrnS of Phraortes sp.. Watson-Crick base pairs are indicated by lines, and wobble G-U base pairs are indicated by dots. The non-canonical base pairs are not marked. The numbers I, II, IV, V and VI represent the five domains in the rrnL gene. The numbers I–Ⅲ represent the three domains in the rrnS gene. (TIF) [file pone.0240186.s002.tif]

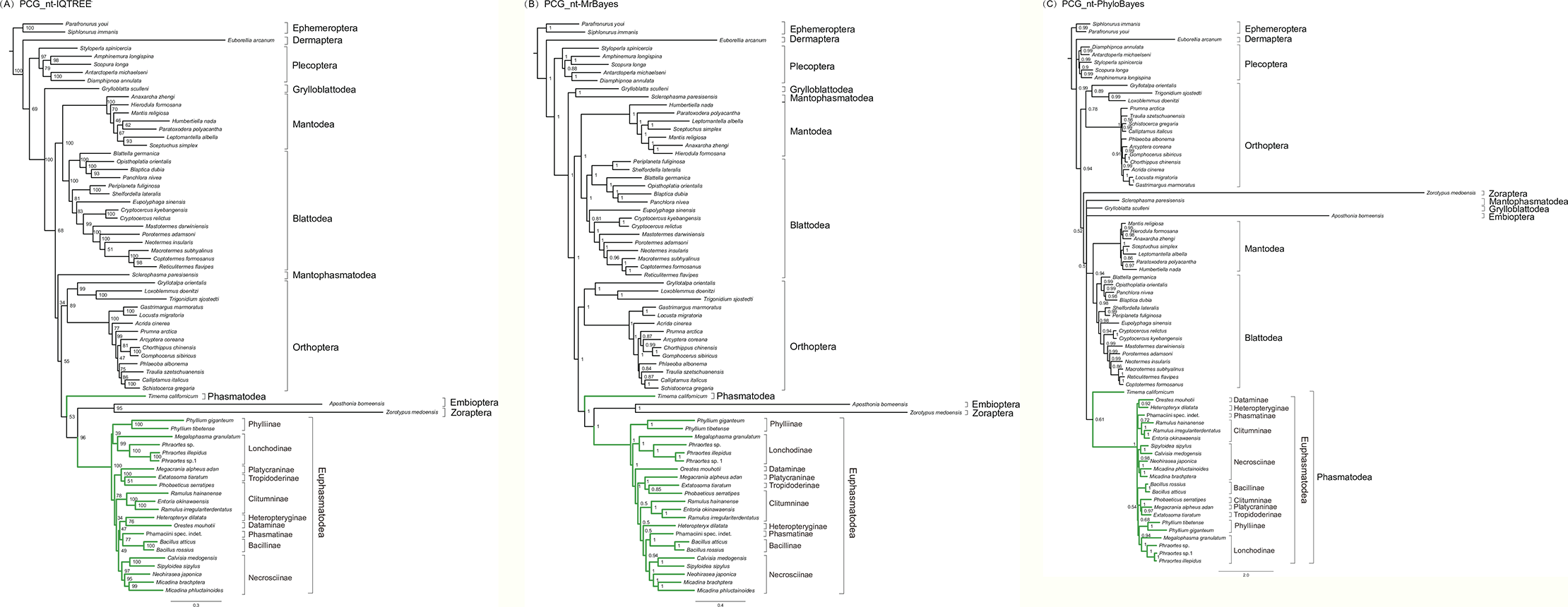

Supplement: S3 Fig — (A) ML tree was reconstructed by IQ-TREE. The partition schemes and best-fitting models were selected by PartitionFinder. Node numbers show bootstrap support values. (B) Bayesian tree was reconstructed by MrBayes. The partition schemes and best-fitting models were selected by PartitionFinder. Node numbers show the poster probability values. (C) Bayesian tree was reconstructed by PhyloBayes. The CAT-GTR model were used in this analysis. Node numbers show the poster probability values. (TIF) [file pone.0240186.s003.tif]

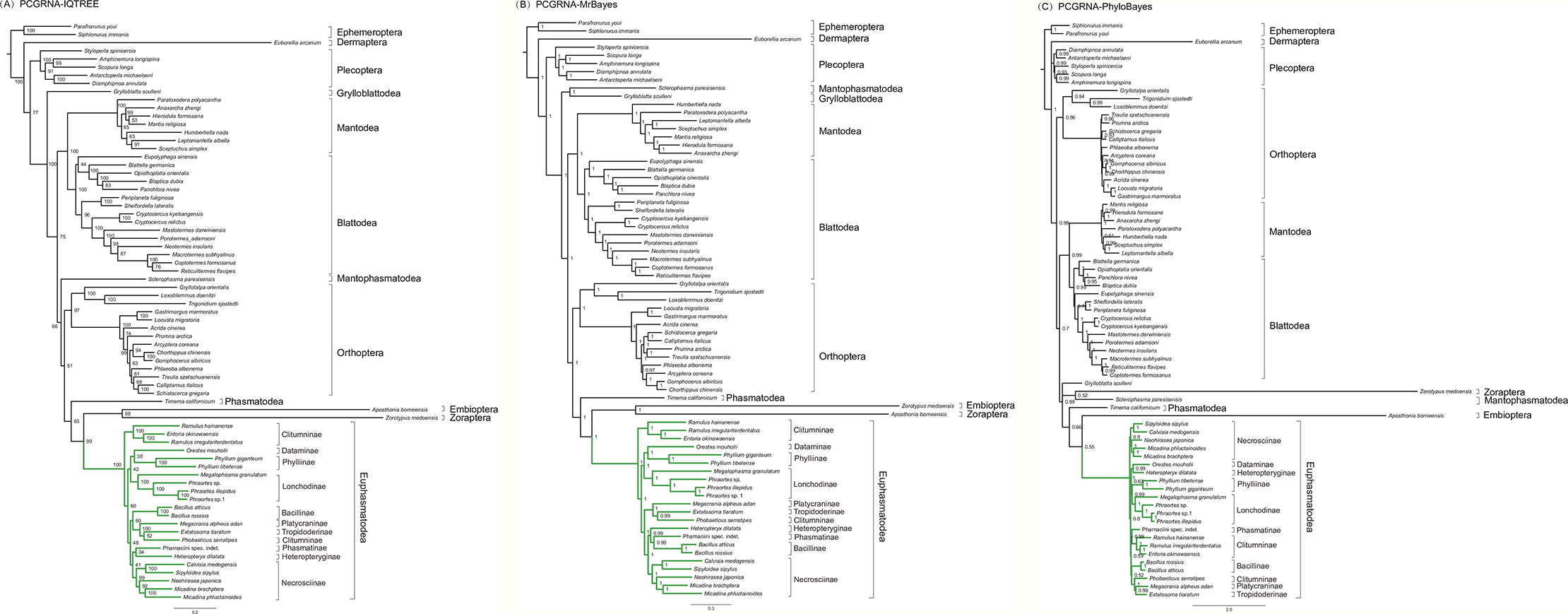

Supplement: S4 Fig — (A) ML tree was reconstructed by IQ-TREE. The partition schemes and best-fitting models were selected by PartitionFinder. Node numbers show bootstrap support values. (B) Bayesian tree was reconstructed by MrBayes. The partition schemes and best-fitting models were selected by PartitionFinder. Node numbers show the poster probability values. (C) Bayesian tree was reconstructed by PhyloBayes. The CAT-GTR model were used in this analysis. Node numbers show the poster probability values. (TIF) [file pone.0240186.s004.tif]

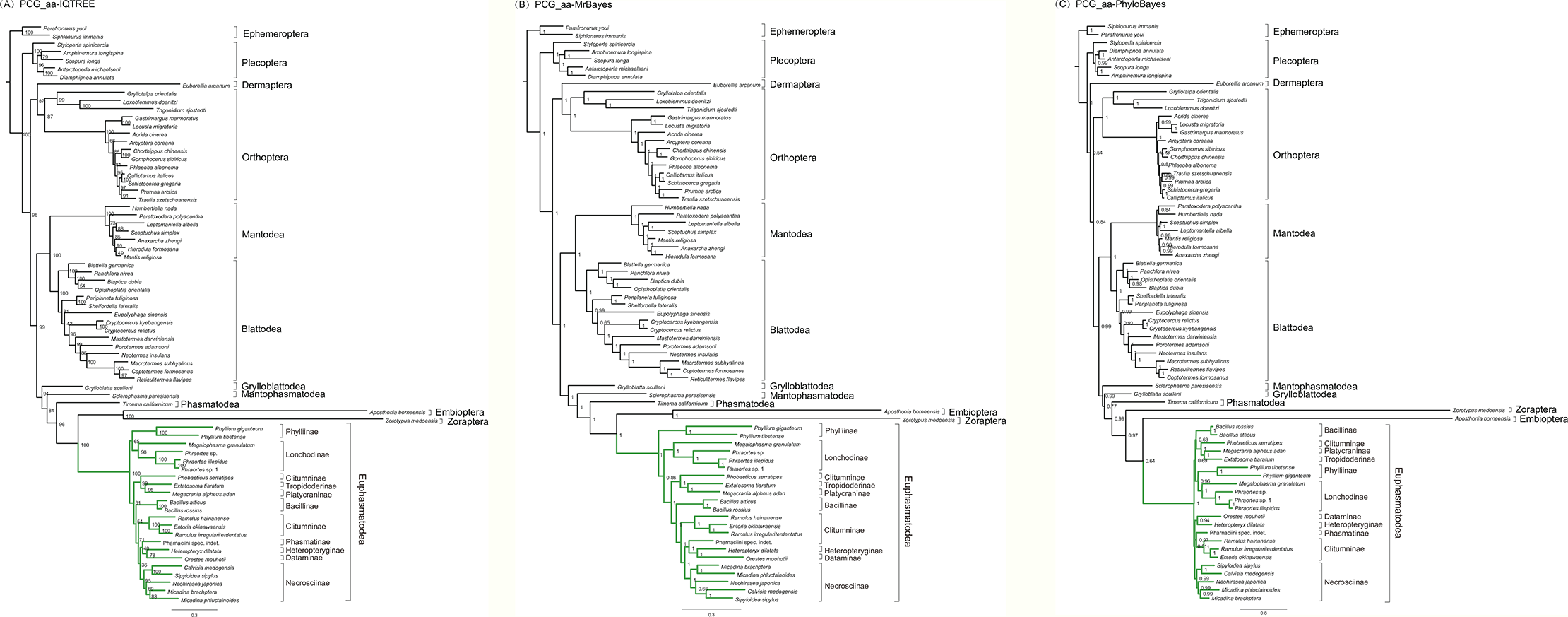

Supplement: S5 Fig — (A) ML tree was reconstructed by IQ-TREE. The partition schemes and best-fitting models were selected by PartitionFinder. Node numbers show bootstrap support values. (B) Bayesian tree was reconstructed by MrBayes. The partition schemes and best-fitting models were selected by PartitionFinder. Node numbers show the poster probability values. (C) Bayesian tree was reconstructed by PhyloBayes. The CAT-MTZOA model were used in this analysis. Node numbers show the poster probability values. (TIF) [file pone.0240186.s005.tif]
